# A Comparison of Iran and UK EQ-5D-3L Value Sets Based on Visual Analogue Scale 

**DOI:** 10.15171/ijhpm.2016.131

**Published:** 2016-09-28

**Authors:** Aliasghar A. Kiadaliri

**Affiliations:** ^1^Clinical Epidemiology Unit, Orthopaedics, Department of Clinical Sciences-Lund, Lund University, Lund, Sweden.; ^2^Health Services Management Research Center, Institute for Futures Studies in Health, Kerman University of Medical Sciences, Kerman, Iran.

**Keywords:** EQ-5D-3L, Visual Analogue Scale (VAS), Iran, UK

## Abstract

**Background:** Preference weights for EQ-5D-3L based on visual analogue scale (VAS) has recently been developed in Iran. The aim of the current study was to compare performance of this value set against the UK VAS-based value set.

**Methods:** The mean scores for all possible 243 health states were compared using Student t test. Absolute agreement and consistency were investigated using concordance correlation coefficient (CCC) and Bland-Altman plot. Health gains for 29 403 possible transitions between pairs of EQ-5D-3L health states were compared. Responsiveness to change and discriminative ability across subgroups of health transitions were assessed.

**Results:** The mean EQ-5D-3L scores were similar for two value sets (mean = 0.31, *P* = 1.00). For 36% of health states, the absolute differences were greater than 0.10. There were three pairwise logical inconsistencies in the Iranian value set. The Iranian scores were lower (higher) for severe (mild) health states than the United Kingdom. The CCC (95% CI) was 0.85 (0.81 to 0.88) and Bland-Altman plot showed good agreement. The mean health gain for all possible transitions predicted by the Iranian value set was higher (0.22 vs. 0.20, *P* < .001) and two value sets predicted opposite transitions in 15% of transitions. The responsiveness of these two value sets were similar with lower discriminative ability for Iranian value set.

**Conclusion:** The Iranian value set attribute lower values to most severe health states and higher values to mild health states compared with the UK value set. Such systematic differences might translate into discrepant health gains and cost-effectiveness which should be taking into account for informed decision-making.

## Introduction

The EQ-5D-3L is a widely used generic preference-based measure to elicit health state utility values for use in cost-utility analyses. It comprises five dimensions: mobility, self-care, usual activities, pain/discomfort, and anxiety/depression. Each dimension has 3 levels: no problems, some problems, extreme problems; resulting in 243 (3^5^) possible health states.^1^ Each health state is assigned an index score by applying a value set elicited from general population or from patients.

There are several valuation techniques to elicit value sets including time trade-off (TTO), standard gamble (SG), visual analogue scale (VAS), person trade-off, and more recently discrete choice experiment (DCE).^2,3^ Among these, the TTO and VAS techniques have commonly been applied to develop the EQ-5D-3L value sets in several countries, especially in Europe.^4^ It should be noted that while VAS is considered as the most feasible valuation techniques, its choice-less nature raise concerns on its ability to elicit strength of preference for health states.^5^ In countries with no national value set, using a value set based on geographic proximity has been suggested^4^ (the UK value set^6^ is the most common in Iran). However, regardless of techniques used to elicit value sets, it has been shown that there might be substantial differences in values across countries and hence developing local value sets have been recommended.^7-11^ In Iran, a VAS-based value set for EQ-5D-3L has recently developed.^12^ The current study aimed to compare the EQ-5D-3L index scores from this newly developed value set^12^ with the UK VAS-base value set.^13^ The results of this study might be of interest to policy-makers in Iran and other developing countries who make decisions on transferring value set from developed countries to their population and its potential impact on economic evaluations.

## Methods

### The EQ-5D-3L Value Sets

The UK VAS-based value set^13^ is based on transformed VAS-based values for 42 EQ-5D-3L health states measured from 2997 eligible respondents (the mean ± standard deviation [SD] age of 47.1 ± 18.1 years, 57% were women, and 31% were current smokers) from the UK general population. The mean absolute difference between observed and the predicted values for these 42 health states was 0.041, with the maximum absolute difference of 0.086. This model include 10 main effect terms, the constant term (a dummy variable if any dimension is at either level 2 or level 3 to capture any deviation from full health), and N3 term (a dummy variable if any dimension is at level 3).

The Iranian VAS-based value set^12^ is based on transformed VAS-based values for the same 42 EQ-5D-3L health states as the UK study^13^ measured from 853 respondents (the mean ± SD age of 38.2 ± 14.7 years, 45% were women, and 14% were current smokers) from city of Tehran (the capital of Iran). The mean absolute difference between observed and the predicted values for these 42 health states was 0.074, with the maximum absolute difference of 0.216.^12^ This model includes 10 main effect terms, the constant term, and I3-squared term (square of number of dimensions at level 3 beyond the first).

### Statistical Analysis

The Iran^12^ and UK^13^ VAS-based EQ-5D-3L index scores for all 243 EQ-5D-3L health states were calculated. Two value sets were compared using Student *t* test, Wilcoxon rank-sum test, and Spearman rank correlation. Agreement between these value sets was evaluated using Bland-Altman plots^14^ and concordance correlation coefficient (CCC) proposed by Lin.^15^ The presence of logical inconsistency (ie, predicting a higher value for a logically worse health state than a logically better health state) was examined. A health state is considered logically better than another health state if it has better status on at least one dimension with no worse status on any other dimension.^16^ Two value sets were compared across five quintiles of health states defined based on the Iranian EQ-5D-3L index scores (from most to least severe health states).

The absolute transition scores in the EQ-5D-3L index scores for 29 403 (_2_C_243_) pairs of EQ-5D health states were compared using Student *t* test. An absolute transition score measures the health utility change for a transition from a worse health state to a better health state.^17^ In addition, the responsiveness of two value sets across consistent health transition (ie, transitions that yield health gain in both value sets) was assessed by assuming the health state with lower value as pre-treatment and the health state with higher value as post-treatment and computing standardized response mean.^17^ Moreover, four possible changes across three levels of EQ-5D-3L were defined: (*i*) major improvement: changes from level 3 to level 1 or 2; (*ii*) minor improvement: changes from level 2 to level 1; (*iii*) minor deterioration: changes from level 1 to level 2; and (*iv*) major deterioration: changes from level 1 or 2 to level 3. Based on these changes, six mutually exclusive subgroups were defined: (1) major improvement with no deterioration, (2) minor improvement with no deterioration, (3) major improvement with minor deterioration, (4) major improvement with major deterioration, (5) minor improvement with minor deterioration, and (6) minor improvement with major deterioration. It should be noted that a transition including both major and minor improvement (deterioration) is considered only as a major improvement (deterioration). In addition, based on the expected health gain for these subgroup, eleven pairwise comparisons were formed and the discriminative ability of two value sets for these pairwise comparisons was assessed by calculating the effect size (the difference between the mean of two subgroups divided by the pooled standard deviation^18^). Due to high number of statistical tests, all *P* values were corrected using Bonferroni correction.

## Results

**Figure 1:**
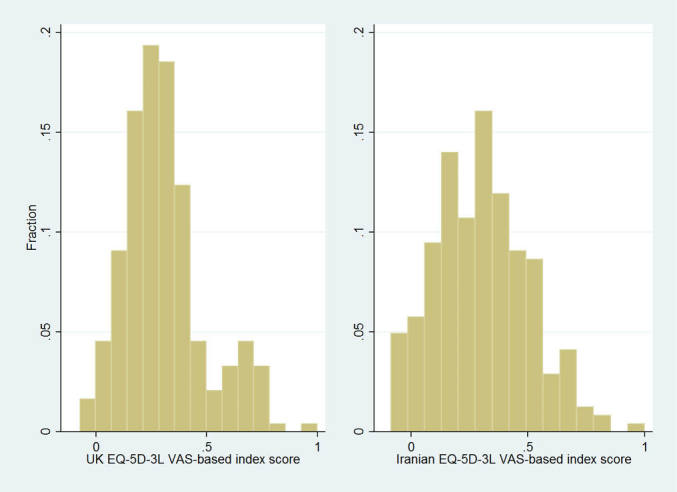
Distribution of EQ-5D-3L VAS-Based Index Scores Predicted by the Iranian and UK Value Sets. Abbreviation: VAS, visual analogue scale.

The mean (SD) of the EQ-5D-3L index score predicted by the Iranian and UK value sets were 0.31 (0.20) and 0.31 (0.18), respectively (Table 1). There were no statistically significant differences in the mean and median of scores predicted by two value sets (*P* = 1.00 for mean and median). Spearman rank correlation between two value sets was 0.87 (*P* < .001). The Iranian value set had a slightly wider range than the UK value set (from −0.09 to 1.00 vs. −0.07 to 1.00, Figure 1). Both value sets attributed a value of 1.00 to health state 11 111. The Iranian value set predicted a lower index score for 129 health states. There were 241 and 187 health states with unique index score in the Iranian and UK value sets, respectively.

**Table 1:** The EQ-5D-3L Index Scores and Absolute Transition Scores Predicted by the Iranian and UK VAS Value Sets

	**n**	**Mean**	**SD**	**Median**	**Minimum**	**Maximum**
EQ-5D-3L index score						
Iran	243	0.31	0.20	0.30	− 0.09	1.00
UK	243	0.31	0.18	0.28	− 0.07	1.00
Iran–UK	243	− 0.00	0.11	− 0.01	− 0.30	0.29
Absolute transitions scores (all transitions)						
Iran	29 403	0.22	0.17		0.00	1.09
UK	29 403	0.20	0.17		0.00	1.07
Iran–UK	29 403	0.02	0.13		− 0.44	0.48

Abbreviation: VAS, visual analogue scale.

The second best health state was “21 111” (index score = 0.80) in the Iranian value set and “11 211” (index score = 0.81) in the UK value set. There were more health states with a negative index scores in the Iranian than UK value set (14 vs. 4). The predicted scores by the Iranian value set for health states 32 333 (−0.09), 23 333 (−0.08), and 22 333 (−0.07) was lower than health state 33 333 (−0.07) implying the presence of three pairwise logical inconsistencies.

**Figure 2:**
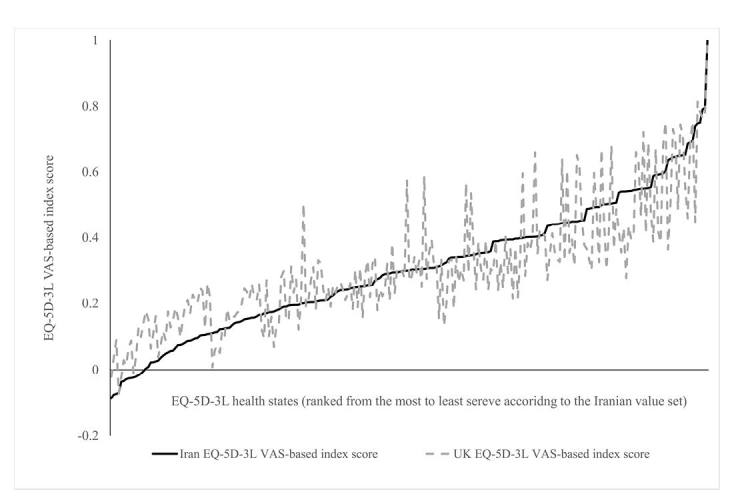
The EQ-5D-3L VAS-Based Index Scores for 243 EQ-5D-3L Health States Predicted by the Iranian and UK Value Sets. Abbreviation: VAS, visual analogue scale.

**Figure 3:**
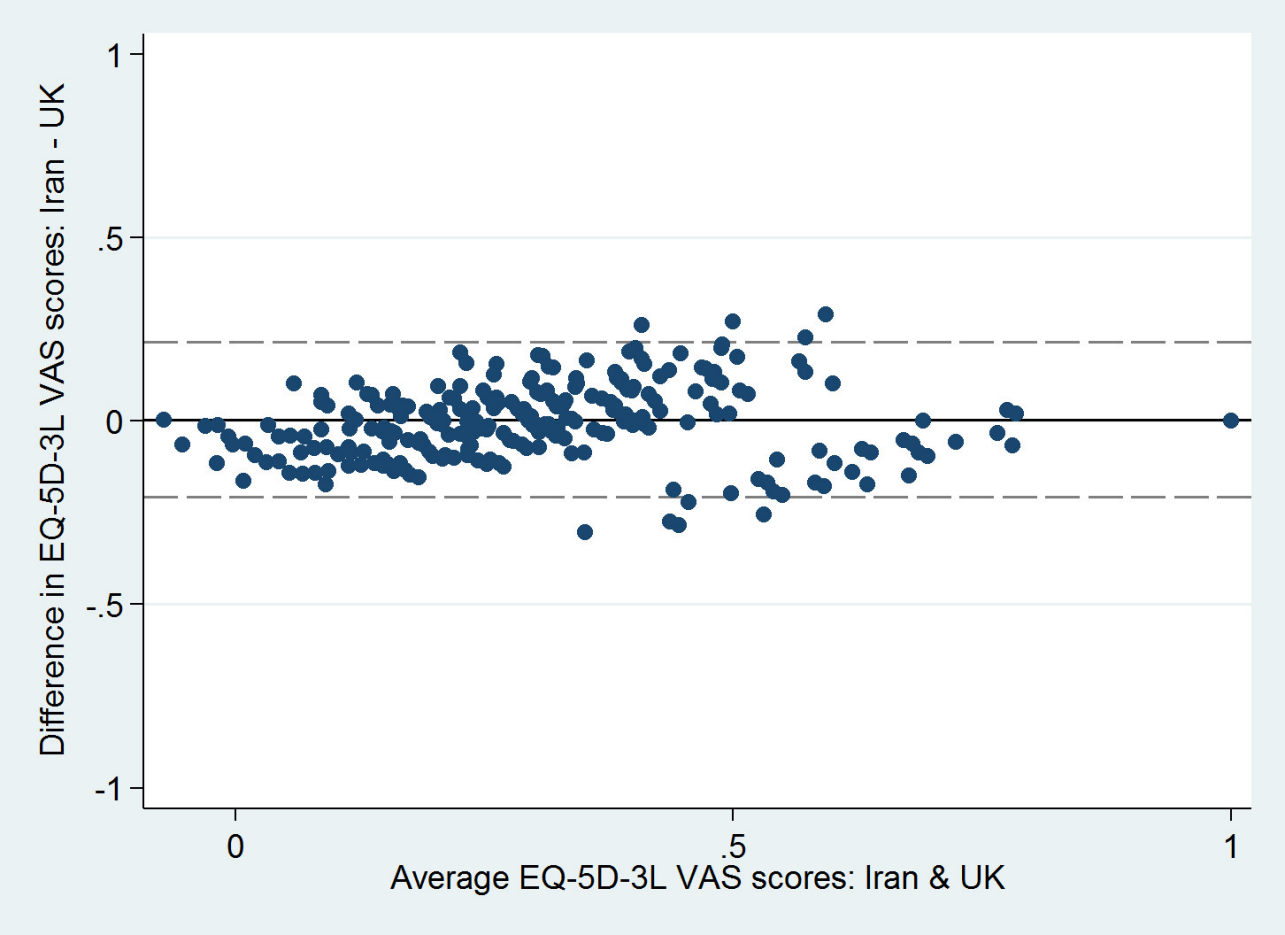
Bland-Altman Plots of EQ-5D-3L VAS-Based Index Scores Predicted by the Iranian and UK Value Sets. Abbreviation: VAS, visual analogue scale.

Across five quintiles of EQ-5D-3L health states, the Iranian value set predicted statistically significantly lower scores for most severe health states and higher scores for least severe health states (Table 2 and Figure 2). The magnitude of absolute difference was higher for mild health states. The Bland-Altman plots (Figure 3) showed that there was a good agreement between two value sets and more than 96% of the differences in EQ-5D-3L index scores fell within the 95% limits of agreement. Agreement between two value sets was good with a CCC (95% CI) of 0.85 (0.81 to 0.88).

**Table 2:** The EQ-5D-3L Index Scores Across Five Quintile of Health States Ranked by The Iranian Value Set

	**Iran, Mean**	**UK, Mean**	**Mean Difference (95% CI)**	**Mean Absolute Difference**
Most severe health states (n = 49)	0.05	0.12	−0.07 (−0.09 to −0.05)	0.08 (0.07 to 0.10)
Q2 (n = 49)	0.19	0.22	−0.03 (−0.05 to −0.01)	0.06 (0.04 to 0.07)
Q3 (n = 48)	0.30	0.29	0.00 (−0.02 to 0.03)	0.07 (0.05 to 0.09)
Q4 (n = 49)	0.41	0.38	0.03 (−0.00 to 0.06)	0.09 (0.07 to 0.11)
Least severe health states (n = 49)	0.60	0.55	0.05 (0.01 to 0.09)	0.12 (0.10 to 0.14)

The mean absolute transition scores for the 29 403 the EQ-5D-3L heath transitions were 0.22 and 0.20 using the Iranian and UK value sets, respectively (mean difference = 0.02, 95% CI: 0.02 to 0.03). In 24 884 (85%) of 29 403 health transitions, both value sets were consistent in predicting health gain/loss. In about 60% of consistent health transitions, the Iranian value set predicted a higher health gain than the United Kingdom with an absolute difference in predicted health gain greater than 0.10 (0.25) in about 46% (7%) of these transitions.

There was a statistically significant difference in health gain predicted by two value sets for consistent health transitions (mean difference = 0.03, *P* < .001) with more profound differences within subgroups of transition (mean difference ranged 0.02 to 0.11, *P* < .001 for all comparisons, Table 3). In all subgroups but “major improvement, minor deterioration” the Iranian value set predicted a higher health gain and had higher responsiveness to change compared with the UK value set.

**Table 3:** Responsiveness of EQ-5D-3L Index Scores Predicted by the UK and Iranian Value Sets Across Consistent Health Transitions

	** All Consistent Transitions (n = 24 884) **		** Major Improvement, no Deterioration (n = 6749) ** ^a^		** Minor Improvement, no Deterioration (n = 781) ** ^b^		** Major Improvement, Minor Deterioration (n = 4969) ** ^c^		** Major Improvement, Major Deterioration (n = 11 407) ** ^d^		** Minor Improvement, Minor Deterioration (n = 509) ** ^e^		** Minor Improvement, Major Deterioration (n = 469) ** ^f^	
	Iran	UK	Iran	UK	Iran	UK	Iran	UK	Iran	UK	Iran	UK	Iran	UK
Pre-treatment EQ-5D-3L index score	0.18	0.20	0.12	0.15	0.27	0.34	0.26	0.24	0.17	0.19	0.38	0.45	0.19	0.24
Post-treatment EQ-5D-3L index score	0.43	0.42	0.49	0.50	0.50	0.46	0.47	0.53	0.38	0.33	0.51	0.52	0.32	0.29
Health gain	0.25	0.22	0.37	0.35	0.23	0.12	0.21	0.29	0.21	0.13	0.13	0.07	0.13	0.05
Standardized response mean	1.49	1.31	2.04	1.92	1.92	1.66	1.49	1.93	1.49	1.47	1.42	1.33	1.41	1.23

^a^ At least one change from level 3 to level 1 or 2, with no deterioration; ^b^ At least one change from level 2 to level 1, with no deterioration; ^c^ At least one change from level 3 to level 1 or 2, with at least one change from level 1 to level 2; ^d^ At least one change from level 3 to level 1 or 2, with at least one change from level 1 or 2 to level 3; ^e^ At least one change from level 2 to level 1, with at least one change from level 1 to level 2; ^f^ At least one change from level 2 to level 1, with at least one change from level 1 or 2 to level 3.

The Iranian value set had generally lower discriminative ability than the UK value set (Table 4) and was not able to discriminate between minor and major deteriorations when the level of improvement was the same (eg, the same health gain for “major improvement with minor deterioration” and “major improvement with major deterioration” while a higher health gain from first subgroup is expected).

**Table 4:** Discriminative Ability of the Iranian and UK Value Sets Across Combinations of Health Transitions

	** Iran Value Set **			**UK Value Set**		
	** Mean Difference **	***P ***	**Effect Size**	** Mean Difference**	*** P***	** Effect Size**
“Major improvement, no deterioration” vs. “minor improvement, no deterioration”	0.14	< .001	0.76	0.24	< .001	1.24
“Major improvement, no deterioration” vs. “major improvement, minor deterioration”	0.16	< .001	0.88	0.06	< .001	0.34
“Major improvement, no deterioration” vs. “major improvement, major deterioration”	0.16	< .001	0.93	0.22	< .001	1.29
“Major improvement, no deterioration” vs. “minor improvement, minor deterioration”	0.24	< .001	1.27	0.29	< .001	1.49
“Major improvement, no deterioration” vs. “minor improvement, major deterioration”	0.24	< .001	1.28	0.31	< .001	1.58
“Minor improvement, no deterioration” vs. “minor improvement, minor deterioration”	0.10	< .001	0.82	0.05	< .001	0.77
“Minor improvement, no deterioration” vs. “minor improvement, major deterioration”	0.10	< .001	0.83	0.07	< .001	1.02
“Major improvement, minor deterioration” vs. “major improvement, major deterioration”	0.00	1.00	0.01	0.16	< .001	1.19
“Major improvement, minor deterioration” vs. “minor improvement, minor deterioration”	0.07	< .001	0.55	0.23	< .001	1.42
“Major improvement, minor deterioration” vs. “minor improvement, major deterioration”	0.08	< .001	0.55	0.25	< .001	1.53
“Major improvement, major deterioration” vs. “Minor improvement, major deterioration”	0.08	< .001	0.55	0.09	< .001	0.96
“Minor improvement, minor deterioration” vs. “Minor improvement, major deterioration”	0.00	1.00	0.01	0.02	< .001	0.41

## Discussion

In the current study, the recently developed Iranian VAS-based EQ-5D-3L valuation was compared with the corresponding valuation in the United Kingdom. The results showed that while there was good overall agreement between two value sets, there were evidence of systematic differences. The Iranian value set predicted lower values for most severe health states and higher values for mild health states. This systematic difference resulted in a higher health gain predicted by the Iranian value set compared with the UK value set and this was more profound for transitions comprise minor improvement with no deterioration.

The higher health gain predicted by the Iranian value set would translate into lower and more favourable incremental cost-effectiveness ratios (ICERs) compared with the UK value set. Of course, in 10 083 (41%) of 24 884 consistent health transitions, the UK value set would result in higher health gain and in turn lower ICERs compared with the Iranian value set. Furthermore, in about 15% of health transitions, two value sets would give completely different results on health gain (ie, for the same transition while one value set predicted a health gain, the other value set predicted a health loss). This figure was about 6% in comparison of the UK and US TTO-based value sets.^17^

Whether different ICERs produced by two value sets translate into discrepant decision funding depends on several factors including the distribution of health transitions in the sample under study, efficacies of interventions, the severity of the health condition under study, cost differences between interventions, and willingness to pay (WTP) threshold.^19^ For example, if health transitions with negligible differences in health gains are more common in a study, then the choice of value set does not influence the estimated ICER. However, if health transitions with substantial differences in health gain are prevalent in a study, then estimated ICER might result in discrepant decisions. This has important policy implications as applying different value sets might generate different results from economic evaluation studies and in turn different decision by policy-makers. Therefore, the impact of the choice of EQ-5D value set on ICERs should be assessed through sensitivity analyses and should be reported to health authorities by healthcare suppliers to aid informed decision-making. In addition, previous healthcare interventions that had been found cost-effective using the UK tariff might not be cost-effective with the Iranian tariff and vice versa.

Interestingly, on all EQ-5D-3L dimensions, (1) a moving from level 1 or 2 to level 3 was associated with higher utility decrement in the UK value set than the Iranian value set, and (2) a moving from level 1 to level 2 had a higher utility decrement than moving from level 2 to level 3 in the Iranian value set, while opposite was observed in the UK value set. Although, the presence of N3 term in the UK value set might seems as an explanation, the similar differences was found in the N3 model specification of the Iranian value set.^12^ In addition, the self-care and anxiety/depression were most important and mobility was least important dimensions of the EQ-5D-3L in the Iranian valuation, while pain and mobility were most important and usual activity was least important dimensions in the UK valuation. These differences might influence the priority given to interventions and should be taking into account by policy-makers. For example, if an intervention influence mainly the mobility dimension of EQ-5D-3L, then priority given to this intervention depends on the value set used (high priority based on the UK value set and low priority base on the Iranian value set). There are several potential explanations for the observed differences between two value sets including inherent differences between two populations (eg, cultural differences) that influenced their valuation, difference in methodologies used to develop value sets, difference induced by translation, time effects (the Iranian value set was recently developed while the UK value set developed in 1995), and difference in response style.^9,20,21^

While application of a national value set is generally supported,^4^ the Iranian value set suffer from several limitations that call for caution in its application. The Iranian valuation study^12^ was conducted in the capital city of Iran with specific health–cultural–socio-economic status which might not be representative of the Iranian general population (eg, highly educated participants of whom 42% had academic education and solely from urban areas). A recent systematic review^22^ showed that education, urbanisation, and healthcare expenditure are associated with utilities attributed to the EQ-5D-3L valuations indicating that the generalizability of the Iranian value set might be limited. In addition, it is not clear on what grounds the final model for the Iranian value set^12^ has been selected. For example, the authors^12^ stated that “all models were tested and compared regarding the number of incoherent coefficients, the statistical significance of the coefficients, the amount of explained variance (R2), the mean absolute error (MAE), and the Akaike information criterion (AIC).” However, the final model did not outperform other specifications based on any of these criteria. Furthermore, the Iranian value set had low discriminative ability to distinguish minor deterioration from major deterioration, and the model^12^ was not validated in an internal or external sample.

The results of the current study should be interpreted in light of several limitations. Due to a lack of data on changes in health status over time, the same probability of occurrence for all health transitions was assumed that is not true in reality. This implies that the results might be different compared to clinical studies where a small subgroup of the EQ-5D-3L health states are present. In addition, a lack of data avoids assessing test-retest reliability of two value sets. Comparing these value sets in longitudinal empirical studies can overcome these limitations.

## Conclusion

While the Iranian and UK value sets provide comparable mean EQ-5D-3L index scores and good agreement, there are systematic differences between two value sets. The Iranian value set attribute lower values for most severe health states and higher values for mild health states than the UK value set. Such systematic differences might translate into discrepant health gains and ICERs which have important policy implications. Moving from level 1 to level 2 of EQ-5D-3L dimensions had more relative importance for the Iranian respondents compared with the UK respondents. The presence of several significant limitations in the Iranian value set including possible sample selection bias and presence of logical inconsistencies implies that it should be applied with caution. In particular, due to logical inconsistency the use of the Iranian value set in samples with severe EQ-5D-3L health states is not recommended. Developing a new value set in Iran using a large representative national survey with a transparent methodology is highly recommended.

## Ethical issues

None. Both Iranian and UK EQ-5D-3L value sets were publicly available and no individual level data were used.

## Competing interests

Author declares that he has no competing interests.

## Author’s contribution

AAK is the single author of the paper.

### Key messages

**Implications for policy makers**

- While the Iranian and UK value sets provide comparable mean EQ-5D-3L index scores and good agreement, there were systematic differences between two value sets.
- Predicting lower values for most severe health states and higher values for mild health states by Iranian value set would result in higher health gain and more favourable cost-effectiveness results for quality of life improving interventions compared with the UK value set.
- Moving from “no problems” to “some problems” on EQ-5D-3L dimensions had more relative importance for the Iranian respondents compared with the UK respondents while the opposite was observed for moving from “some problems” to “extreme problems.”
- Health authorities should be aware of the potential impact of different value sets on cost-effectiveness analyses, especially since it is probable that healthcare supplier applies a value set which supports their products.

**Implications for public**

Comparing the Iranian- and UK-VAS based EQ-5D-3L index scores showed that there were systematic differences between these two value sets implying that the UK value set might not be applicable for the Iranian population. However, due to possible sample selection bias, the presence of logical inconsistencies, and low know-group validity for health transitions, it is suggested that the Iranian value set should be applied with caution.
